# Effects of different *Lactobacillus plantarum* strains supplementation on milk performance, rumen fermentation, and microbiota in dairy cows

**DOI:** 10.3389/fmicb.2026.1774000

**Published:** 2026-03-10

**Authors:** JiYou Zhang, Han Zhang, Hongwei Duan, Fuzhen Zhou, Weiming Xiao

**Affiliations:** 1 Key Laboratory of Dairy Cow Genetic Improvement and Milk Quality Research of Zhejiang Province, Zhejiang Zhongxing Animal Husbandry Technology Co., Ltd., Wenzhou, China; 2 Ministry of Education Key Laboratory of Molecular Animal Nutrition, Zhejiang University, Hangzhou, China; 3 Ruminant Nutrition and Feed Engineering Technology Research Center, College of Animal Science and Technology, Nanjing Agricultural University, Nanjing, China

**Keywords:** dairy cows, *Lactobacillus plantarum*, milk performance, rumen bacterial community, rumen fermentation

## Abstract

This study combined *in vitro* and *in vivo* experiments to evaluate the effects of three *Lactobacillus plantarum* strains (AP 6-5, 18-5-5, and Y2-2-3) on rumen fermentation, microbiota composition, and productive performance in lactating Holstein cows. In the *in vitro* trial, rumen fluid from three fistulated cows was incubated with TMR substrate supplemented with each strain (1 × 10^7^ CFU/mL) at 39 °C for 24 h. Compared with the CON, strains AP 6-5 and 18-5-5 reduced ruminal lactate and acetate concentrations, and strain 18-5-5 further decreased microbial crude protein (MCP). In the *in vivo* trial, forty-four cows were randomly assigned to four groups: a control (CON) and three treatment groups receiving 5 × 10^10^ CFU/d of strains AP 6-5 (AJT), 18-5-5 (NM), or Y2-2-3 (LP42) for 28 days after a 7-day adaptation. The NM group showed the highest DMI, milk yield, and lactose content, while the LP42 group had higher valeric acid concentration and fecal pH. Rumen microbiota analysis indicated enrichment of pathways related to carbohydrate utilization (NM) and protein metabolism (LP42). Overall, supplementation with *L. plantarum*, particularly strain 18-5-5, improved nutrient intake, milk production, and rumen microbial function in lactating cows.

## 1 Introduction

The increasing demand for high milk yield in dairy cows requires nutritional strategies that not only maximize productivity but also maintain rumen stability, animal health, and farm sustainability (VandeHaar and St-Pierre, 2006). In lactating dairy cows, the rumen functions as a highly specialized anaerobic fermentation chamber, harboring a complex microbial ecosystem composed of bacteria, protozoa, fungi, and archaea. These microorganisms convert dietary carbohydrates into volatile fatty acids (VFA), which provide the primary source of metabolizable energy for the host (Reddy and Hyder, 2023). Therefore, regulation of rumen microbial composition and fermentation efficiency is essential for improving milk production and metabolic performance.

Probiotics have gained considerable attention as dietary additives capable of modulating gastrointestinal microbiota and improving host productivity. Among them, lactic acid bacteria (LAB) are widely recognized for their probiotic functions, including regulation of microbial balance, enhancement of nutrient digestibility, and modulation of immune responses (Tsai et al., 2012). Species belonging to the genus *Lactobacillus* have been reported to modulate rumen fermentation, enhance feed utilization, and promote overall health and performance in ruminants (Chaucheyras-Durand and Durand, 2010). In particular, *Lactobacillus plantarum*, a widely used probiotic lactic acid bacterium, has been shown to regulate gut microbial balance, improve nutrient digestibility, and strengthen immune responses in ruminants (Wang et al., 2018). Previous studies have demonstrated that dietary supplementation with *L. plantarum* can enhance milk yield and composition in dairy cows, largely through its effects on rumen fermentation. Monteiro et al. (2021) reported that cows supplemented with *L. plantarum* MTD-1 produced more milk, likely due to improved dry matter intake and organic matter digestibility. Such benefits are attributed to the ability of *L. plantarum* to stimulate VFA production, thereby increasing energy availability to the host (O'Hara et al., 2020). In addition, several studies have evaluated the potential of different *Lactobacillus* species to enhance rumen function and milk performance in dairy cows (Boyd et al., 2011; Monteiro et al., 2022; Philippeau et al., 2017; Raeth-Knight et al., 2007).

However, probiotic effects are often strain-specific. Different *L. plantarum* strains may vary in their ability to survive in the rumen environment, adhere to mucosal surfaces, modulate immune responses, and alter rumen microbial communities (Campana et al., 2017). Despite the recognized probiotic potential of *L. plantarum*, comparative information on the strain-specific effects of isolates such as 18-5-5, AP 6-5, and Y2-2-3 on rumen fermentation, microbial composition, and milk performance in dairy cows remains limited. Therefore, the present study aimed to evaluate the effects of dietary inclusion of *L. plantarum* strains 18-5-5, AP 6-5, and Y2-2-3 on rumen fermentation characteristics, microbiota composition, blood biochemical indices, and milk yield in lactating Holstein cows.

## 2 Materials and methods

### 2.1 Animals and experimental design

This study consisted of two experiments: an *in vitro* fermentation experiment and an *in vivo* feeding experiment. The experiment included four groups: a control group (CON) without probiotic supplementation and three treatment groups receiving 5 × 10^10^ CFU/d of *Lactobacillus plantarum* strains AP 6-5 (AJT), 18-5-5 (NM), and Y2-2-3 (LP42), respectively. In *in vitro* experiment, three strains of *Lactobacillus plantarum* were selected for the *in vitro* fermentation experiment to evaluate their effects on ruminal fermentation. Each strain was added to the incubation medium at a final concentration of 1 × 10^5^ CFU/mL. Each treatment had three parallel samples, and the entire experiment was repeated three times. Based on the results of the *in vitro* experiment, the supplementation level was adjusted according to practical dairy production conditions to conduct an *in vivo* feeding experiment, aiming to further verify the in vitro findings.

All cows used in both experiments were obtained from Zhenjiang Ruzhiyuan Ecological Animal Husbandry Co., Ltd. (Zhenjiang, Jiangsu, China). Cows were milked and fed three times daily (0500, 1,300, and 2000) and were offered a total mixed ration (TMR) formulated according to the nutrient requirements of dairy cows recommended by NASEM (2021) (Table 1). Feed was supplied to allow 5–10% refusals. During the experiment, cows were individually tied in indoor stalls with free access to clean drinking water. The *L. plantarum* strains were isolated and purified by the Ruminant Innovation Team of Nanjing Agricultural University, and the bacterial suspension contained 1 × 10^7^ CFU/mL of viable cells. In the *in vivo* experiment, the bacterial suspension was mixed with the TMR at a specific proportion, and the detailed dosage is described below.

**Table 1:** Nutrient composition of experimental diet (DM, %).

Feed ingredients	Composition
Corn	19.33
Brewer’s grains	10.03
Corn Silage	28.08
Leymus chinensis	5.58
Alfalfa	11.47
Soybean meal	11.03
Bran	2.78
DDGS	4.39
Molasses bean curd	2.83
Salt	0.55
baking soda	0.83
Premix^1^	2.25
Mold remover	0.06
Calcium Hydrogen Phosphate	0.59
Dry yeast	0.19
Nutritional content	
DM	44.63
CP	15.87
NDF	31.75
ADF	17.26
Ash	7.76

^1^Per kilogram of premix provided: selenium (from selenium yeast), 7 mg; selenium (from sodium selenite), 6 mg; iodine, 20 mg; cobalt, 4 mg; copper, 360 mg; iron, 170 mg; manganese, 910 mg; zinc (elemental), 680 mg; methionine hydroxy analogue, 10 g; sodium chloride, 11.18%; total phosphorus, 1.0%; calcium, 5.1%; magnesium, 4.0%; vitamin A, 180,000 IU; vitamin D₃, 45,000 IU; and vitamin E, 1,400 IU.

#### 2.1.1 *In vitro* fermentation

Before morning feeding, rumen fluid was collected via an oral stomach tube from three lactating dairy cows and immediately transferred to the laboratory in pre-warmed insulated flasks. In the laboratory, the rumen fluid was filtered through four layers of cheesecloth under continuous CO₂ flushing and maintained at 39 °C in a water bath. The filtrate was then mixed with artificial saliva at a ratio of 1:2 (v/v), and the preparation of artificial saliva following the method of Theodorou et al. (1994). For incubation, 49.5 mL of the mixed fermentation medium was dispensed into 100 mL serum bottles, to which 1 g of TMR substrate and 0.5 mL of bacterial suspension were added. The control (CON) group received 0.5 mL of distilled water instead. During dispensing, continuous CO₂ was flushed to maintain anaerobic conditions. The bottles were immediately sealed with butyl rubber stoppers and aluminum caps and incubated at 39 °C for 24 h. After incubation, total gas production was measured using an air pressure transducer, and gas samples were collected in gas bags for methane determination. Fermentation was then terminated by immersing the bottles in ice water. The pH of the fermentation fluid was measured using a portable pH meter, and aliquots were collected for the determination of NH₃-N, VFA, and microbial crude protein (MCP).

Rumen fluid samples (1 mL) were mixed with 0.2 mL of 25% (w/v) orthophosphoric acid and analyzed for volatile fatty acid (VFA) concentrations using gas chromatography (GC-14B, Shimadzu, Japan) as described by Zhang et al. (2022). The concentration of ammonia nitrogen (NH₃–N) in rumen fluid was determined colorimetrically according to the method of Chaney and Marbach (1962). Microbial crude protein (MCP) concentration was determined following previously described procedures.

#### 2.1.2 *In vivo* feeding experiment

Based on the *in vitro* results, the effective concentration of *Lactobacillus plantarum* was determined as 1 × 10^5^ CFU/mL. Considering the rumen volume of dairy cows (approximately 150 L) and a feeding frequency of three times per day, the corresponding *in vivo* supplementation dosage was calculated to be 500 mL of bacterial suspension per cow per day, with a viable count of 1 × 10^7^ CFU/mL.

A total of 44 multiparous Holstein cows with similar body condition, parity (2–3), milk yield (Mean ± SD; 28.1 ± 1.8 kg/d), and days in milk (158 ± 16 d) were selected and randomly assigned to four dietary treatments using a completely randomized block design (*n* = 11 per group). The control (CON) group received 500 mL of distilled water, whereas the treatment groups were supplemented with equal volumes of *L. plantarum* suspensions of different strains. The bacterial suspension was mixed into the total mixed ration (TMR) during feeding. The experiment consisted of a 7-day adaptation period followed by a 28-day feeding period.

### 2.2 Sampling and measurement

#### 2.2.1 Production performance

During the feeding experiment, milk yield of each cow was automatically recorded weekly using an automated sampling system (Waikato Milking Systems Ltd., Hamilton, New Zealand). Milk samples were collected at each milking in a 4:3:3 ratio to obtain a 50 mL composite sample. Potassium dichromate was added as a preservative, and samples were stored at 4 °C until analysis. Milk composition, including fat, protein, lactose, total solids, milk urea nitrogen (MUN), and somatic cell count (SCC), was analyzed at the Dairy Herd Improvement (DHI) Center of Nanjing Agricultural University.

#### 2.2.2 Feed composition and nutrient digestibility

Dry matter intake (DMI) was recorded daily and averaged weekly during the feeding experiment. Representative samples (500 g) of the offered TMR and refusals were collected weekly and stored at −20 °C for chemical analysis. The samples were oven-dried at 65 °C for 48 h to a constant weight to determine dry matter (DM) content. Dried samples were then ground to pass through a 1-mm screen (40 mesh) and stored at −20 °C until analysis. Crude protein (CP), ether extract (EE), neutral detergent fibre (NDF), and acid detergent fibre (ADF) were analyzed according to the procedures of AOAC (2005). The apparent digestibility was calculated using acid-insoluble ash (AIA) as a marker, following the methods of Van Keulen and Young, 1977. The calculation formula is as follows:

$$\begin{array}{l} \text{Nutrient}\;\\ \text{digestibility}\;(\%)=[\begin{array}{c} 1-(\begin{array}{l} \mathrm{AIA}\;\text{concentration in feed}/\\ \mathrm{AIA}\;\text{concentration in feces} \end{array})\times \\ \left(\begin{array}{l} \text{Nutrient concentration in feces}/\\ \text{Nutrient concentration in feed} \end{array}\right) \end{array}]\times 100\% \end{array}$$

#### 2.2.3 Blood collection and analysis

On day 27 of the feeding experiment, blood samples (10 mL) were collected from the coccygeal vein into vacuum tubes containing sodium heparin, 1 h before the morning feeding. The samples were centrifuged at 3500 rpm for 15 min at 25 °C to obtain plasma, which was stored at −20 °C until further analysis. Plasma biochemical parameters, including glucose, total protein, albumin, blood urea nitrogen (BUN), triglyceride (TG), total cholesterol (T-CHO), aspartate aminotransferase (AST), and alanine aminotransferase (ALT), were determined using an automatic biochemical analyzer (Hitachi 7,020, Hitachi High-Technologies Corporation, Tokyo, Japan).

#### 2.2.4 Rumen fluid collection and analysis

On day 28 of the feeding experiment, rumen fluid was collected 4 h after the morning feeding using an oral stomach tube as described by Shen et al. (2012). Rumen pH was immediately measured using a portable pH meter (HANNA Instruments, Woonsocket, USA). The rumen contents were filtered through four layers of sterile cheesecloth to remove large feed particles. The filtrate was then aliquoted into sterile centrifuge tubes (four 5 mL and two 10 mL portions) and stored at −20 °C until analysis. Stored rumen fluid samples were analyzed for NH₃–N, VFA, and MCP.

#### 2.2.5 Bacterial DNA extraction

Rumen bacterial DNA was extracted using the bead-beating method as described by Mao et al. (2012). Approximately 200 mg of rumen content from each cow was transferred into individual microtubes. After adding lysis buffer, the samples were subjected to vigorous shaking on a mini-bead beater (Biospec Products, Bartlesville, OK, USA) for 5 min. Phenol-chloroform extraction followed by ethanol precipitation was performed to isolate DNA, and the resulting DNA pellets were dissolved in EDTA buffer. DNA purity and concentration were measured using an ND-1000 spectrophotometer (Thermo Fisher Scientific, Waltham, MA, USA), and the samples were stored at −80 °C until further use for amplification and sequencing.

#### 2.2.6 DNA amplification and sequencing

DNA amplification and sequencing were performed following the protocol described by Xie et al. (2021). The V3–V4 region of the bacterial 16S rRNA gene was amplified with primers 341F (5’-CCTAYGGGRBGCASCAG-3′) and 806R (5’-GGACTACNNGGGTATCTAAT-3′), and the resulting amplicons were purified with a QIAquick PCR Purification Kit (Qiagen, Hilden, Germany). Sequencing was carried out on an Illumina MiSeq PE250 platform (Illumina Inc., San Diego, CA, USA). Low-quality reads and adapter sequences were removed using Trimmomatic v0.33 (Bolger et al., 2014). The resulting clean reads were processed with QIIME v1.9.0 (Caporaso et al., 2010), and paired-end reads were merged into tags using FLASH v1.2.7 (Magoc and Salzberg, 2011). The tags were then clustered into operational taxonomic units (OTUs) at 97% sequence similarity using UPARSE v7.0.1001 (Edgar, 2013). Representative sequences from each OTU were taxonomically assigned by comparison with the SILVA 132 database (Quast et al., 2013). Functional prediction of microbial communities was performed using Tax4Fun2 (Wemheuer et al., 2020) implemented in R based on the Ref99NR reference database from the National Center for Biotechnology Information (NCBI).

### 2.3 Statistical analysis

Data on blood biochemistry and rumen fermentation parameters were analyzed using one-way ANOVA in IBM SPSS Statistics (version 20.0). Milk yield, milk composition, and dry matter intake (DMI) data were analyzed using the general linear model (GLM) procedure of SAS (version 9.4; SAS Institute Inc., Cary, NC, USA). Differences among treatments were considered significant at *p* < 0.05. For microbial data, the 16S rRNA sequencing results and predicted KEGG pathways were filtered to retain features with a relative abundance ≥ 0.1% in at least one sample. The Linear Discriminant Analysis Effect Size (LEfSe) method (Segata et al., 2011) implemented in the Microeco package in RStudio (Liu et al., 2020) was used to identify taxa and pathways with differential abundance. Significance thresholds were set at *p* < 0.05 and linear discriminant analysis (LDA) scores ≥ 2.5 for bacterial genera, ≥ 2.7 for phyla, and ≥ 3.0 for KEGG pathways. Alpha diversity indices, principal coordinate analysis (PCoA) based on Bray–Curtis distance, and permutational multivariate analysis of variance (PERMANOVA) were computed using the vegan and Microeco packages in RStudio.

## 3 Results

### 3.1 *In vitro* rumen fermentation and methane production

The effects of different *Lactobacillus* strains on *in vitro* rumen fermentation and methane production are summarized in Table 2. Significant differences (*p* < 0.05) were observed in lactic acid, microbial protein (MCP), and acetic acid concentrations, whereas methane production, pH, ammonia nitrogen, and other VFAs remained unaffected. Lactic acid concentration was highest in the CON and LP42 groups, followed by AJT and NM (*p* < 0.01). The concentration of MCP was significantly lower in NM (46.15 ng/mL) than in the other treatments (*p* = 0.002). The concentration of acetate decreased markedly (*p* = 0.034) in all treatment groups compared with the CON group, while no significant differences were observed in the concentrations of propionate, butyrate, isobutyrate, isovalerate, or valerate (*p* > 0.05).

**Table 2:** *In vitro* VFA and gas production from rumen fluid with different *Lactobacillus* strains.

Item	Treatment				SEM	*P*-value
	CON	AJT	NM	LP42		
Methane production (mL/g DM)	85.19	86.42	85.75	86.22	2.422	0.139
pH	6.02	6.03	6.04	6.04	0.004	0.317
Lactate (mg/mL)	0.43 ^a^	0.38 ^b^	0.35 ^c^	0.41 ^a^	0.006	<0.001
NH_3_-N (mg/dL)	15.36	15.41	15.53	16.02	0.115	0.155
MCP (ng/mL)	46.36 ^a^	46.41^a^	46.15 ^b^	46.37 ^a^	0.031	0.002
Acetate (mM)	58.56 ^a^	46.12 ^b^	48.00 ^b^	48.86 ^b^	1.128	0.034
Propionate (mM)	27.86	25.14	26.71	28.85	0.382	0.171
Isobutyrate (mM)	5.15	4.68	4.94	5.36	0.132	0.263
Butyrate (mM)	12.49	11.72	12.40	13.38	0.338	0.307
Isovalerate (mM)	2.91	3.09	3.21	3.53	0.095	0.197
Valerate (mM)	2.66	2.49	2.55	2.84	0.075	0.289

^a,b^Means within the same row with different superscripts differ significantly (*p* < 0.05).

NH_3_-N, ammonia nitrogen; MCP, microbial protein.

### 3.2 Production performance

The effects of dietary supplementation with different *Lactobacillus* strains on feed intake, body weight, and milk performance of Holstein cows are presented in Table 3. Dry matter intake (DMI) differed significantly among treatments (*p* = 0.008), whereas no differences were observed in body weight. Cows in the NM group had the highest DMI (23.85 kg/day), which was significantly greater than those in the AJT (22.61 kg/day) and CON (23.39 kg/day) groups.

**Table 3:** Effects of different *Lactobacillus* strains on production performance in dairy cows.

Item	Treatment^1^				SEM	*P*-value		
	CON	AJT	NM	LP42		Trt	week	Trt*week
DMI (kg/cow/d)	23.39^c^	22.61^b^	23.85^a^	23.25^ab^	0.177	0.008	-	-
BW (kg)	608.86	613.57	606.18	607.21	10.620	0.995	-	-
Milk yield (kg/d)	27.95^c^	30.74^b^	33.57^a^	32.21^ab^	0.430	<0.001	0.451	0.819
Milk fat (%)	4.40^a^	4.24^ab^	4.13^b^	4.07^b^	0.047	0.047	<0.001	0.355
Milk protein (%)	3.75^a^	3.66^a^	3.58^a^	3.54^a^	0.034	0.137	0.126	0.616
Lactose (%)	4.75^b^	4.82^a^	4.85^a^	4.80^ab^	0.013	0.019	<0.001	0.907
Total solid (%)	14.20^a^	13.58^ab^	12.81^c^	13.11^bc^	0.152	0.001	<0.001	0.259
Urea nitrogen (mg/dl)	16.05^a^	15.94^a^	16.64^a^	16.88^c^	0.171	0.124	<0.001	0.700
SCC (1,000/ml)	286.00^a^	153.50^a^	244.50^a^	436.50^a^	53.700	0.317	0.589	0.585
Solid non-fat (%)	9.60^a^	9.57^a^	9.49^a^	9.43^a^	0.034	0.237	<0.001	0.552

^a,b^Means within the same row with different superscripts differ significantly (*P* < 0.05).

^1^Treatment: CON = basal diet; AJT = basal diet + *Lactobacillus plantarum* AP 6-5 (5 × 10^10^ CFU/d); NM = basal diet + *Lactobacillus plantarum* 18-5-5 (5 × 10^10^ CFU/d); LP42 = basal diet + *Lactobacillus plantarum* Y2-2-3 (5 × 10^10^ CFU/d).

DMI, dry matter intake; BW, body weight; SCC, somatic cell count.

Milk yield and composition were also affected by the dietary treatments (*p* < 0.05). The NM group produced the highest milk yield (33.57 kg/day), significantly exceeding the CON (27.95 kg/day) and AJT (30.74 kg/day) groups. Milk fat percentage was greatest in the CON group (4.40%), significantly higher than in the NM (4.13%) and LP42 (4.07%) groups (*p* = 0.047). The lactose content was highest in the NM group (4.85%), which was significantly greater than in the CON group (4.75%) but comparable to AJT and LP42. Total solids also varied among treatments (*p* = 0.001), with the CON group showing the highest value (14.20%), followed by AJT (13.58%), NM (12.81%), and LP42 (13.11%).

### 3.3 Rumen fermentation and nutrient apparent digestibility

The effects of different *Lactobacillus* strains on rumen fermentation parameters and nutrient digestibility are summarized in Tables 4, 5. Significant differences (*p* < 0.05) were observed in fecal pH and the concentration of valeric acid, whereas other fermentation parameters remained unaffected (*p* > 0.05). Cows in the NM (6.53) and LP42 (6.56) groups showed higher fecal pH values compared with the CON (6.32) and AJT (6.37) groups. The LP42 group also exhibited the highest valeric acid concentration (1.18), which was significantly greater than in the CON and NM groups.

**Table 4:** Effects of different *Lactobacillus* strains on rumen fermentation and fecal pH in dairy cows.

Item	Treatment^1^				SEM	*P*-value
	CON	AJT	NM	LP42		
Rumen pH	6.34	6.30	6.42	6.43	0.036	0.590
Fecal pH	6.32^b^	6.37^b^	6.53^a^	6.56^a^	0.031	0.004
NH_3_-N (mg/dL)	20.67	16.86	15.88	18.74	0.774	0.122
MCP (ng/mL)	203.96	199.75	220.75	229.31	12.579	0.845
Acetate (mM)	75.92	70.02	67.89	74.98	1.453	0.144
Propionate (mM)	21.85	21.20	19.77	23.26	0.588	0.209
Isobutyrate (mM)	0.95	0.68	0.63	0.81	0.086	0.606
Butyrate (mM)	10.86	12.51	10.83	12.70	0.597	0.571
Isovalerate (mM)	0.60	1.06	0.55	0.89	0.087	0.102
Valerate (mM)	0.73^b^	1.10^ab^	0.70^b^	1.18^a^	0.079	0.039

^a,b^Means within the same row with different superscripts differ significantly (*P* < 0.05).

^1^Treatment: CON = basal diet; AJT = basal diet + *Lactobacillus plantarum* AP 6-5 (5 × 10^10^ CFU/d); NM = basal diet + *Lactobacillus plantarum* 18-5-5 (5 × 10^10^ CFU/d); LP42 = basal diet + *Lactobacillus plantarum* Y2-2-3 (5 × 10^10^ CFU/d).

NH_3_-N, ammonia nitrogen; MCP, microbial protein.

**Table 5:** Effects of different *Lactobacillus* strains on apparent nutrient digestibility in dairy cows.

Item	Treatment^1^				SEM	*P*-value
	CON	AJT	NM	LP42		
CP (%)	80.39	78.91	80.97	79.95	0.543	0.624
NDF (%)	64.92	57.01	60.67	57.62	1.674	0.339
ADF (%)	63.15	54.77	58.60	55.29	1.774	0.334
EE (%)	73.01	68.44	70.82	66.98	1.343	0.43

^1^Treatment: CON = basal diet; AJT = basal diet + *Lactobacillus plantarum* AP 6-5 (5 × 10^10^ CFU/d); NM = basal diet + *Lactobacillus plantarum* 18-5-5 (5 × 10^10^ CFU/d); LP42 = basal diet + *Lactobacillus plantarum* Y2-2-3 (5 × 10^10^ CFU/d).

CP, crude protein; NDF, neutral detergent fiber; ADF, acid detergent fiber; EE, ether extract.

Apparent nutrient digestibility was not significantly influenced by the dietary inclusion of different *Lactobacillus* strains, as no statistical differences were detected among treatments for any of the measured parameters.

### 3.4 Blood biochemical indices

Table 6 presents the plasma biochemical indices of cows fed different strains of *Lactobacillus* (NM, AJT, and LP42) compared with the control (CON). The concentrations of total cholesterol (TCHO) and high-density lipoprotein cholesterol (HDL-C) differed significantly among the groups (*p* < 0.05), whereas all other blood biochemical parameters showed no significant differences (*p* > 0.05). Cows supplemented with the AJT strain had the highest TCHO concentration (5.86 mmol/L), which was significantly greater than that of the CON (4.63 mmol/L) and NM (4.40 mmol/L) groups. Similarly, the AJT group exhibited the highest HDL-C concentration (2.58 mmol/L), followed by LP42 (2.34 mmol/L); both values were significantly higher than those of the CON (2.05 mmol/L) and NM (2.19 mmol/L) groups.

**Table 6:** Effects of different *Lactobacillus* strains on blood biochemical indices in dairy cows.

Item	Treatment^1^				SEM	*P*-value
	CON	AJT	NM	LP42		
AST (U/L)	87.00	92.20	84.60	86.60	2.47	0.762
ALT (U/L)	30.60	42.40	36.40	35.60	1.91	0.187
TP (g/L)	79.34	79.53	75.53	82.46	1.53	0.488
ALB (g/L)	32.46	33.18	33.08	32.90	0.52	0.970
GLOB (g/L)	46.90	46.36	42.44	49.54	1.53	0.462
A/G	0.72	0.76	0.78	0.66	0.02	0.279
ALP (U/L)	49.40	62.40	55.00	55.60	3.39	0.638
GGT (U/L)	23.00	26.80	27.60	27.20	1.56	0.741
TBA (μmol/L)	39.48	49.04	36.88	43.80	4.41	0.807
Urea (mmol/L)	5.37	5.75	4.99	5.81	0.17	0.289
CREA (μmol/L)	53.48	55.64	47.38	49.52	1.47	0.184
GLU (mmol/L)	3.41	3.21	3.41	3.34	0.07	0.763
CO_2_ (mmol/L)	27.22	23.76	25.78	24.80	0.66	0.308
NO (μmol/L)	10.40	7.75	14.40	12.60	1.23	0.291
TCHO (mmol/L)	4.63 ^b^	5.86 ^a^	4.40 ^b^	5.32 ^ab^	0.204	0.031
TRIG (mmol/L)	0.11	0.12	0.12	0.11	0.007	0.980
HDL-C (mmol/L)	2.05 ^b^	2.58 ^a^	2.19 ^b^	2.34 ^ab^	0.07	0.041
LDL-C (mmol/L)	1.51	1.73	1.23	1.63	0.09	0.263

^a,b^Means within the same row with different superscripts differ significantly (*P* < 0.05).

^1^Treatment: CON = basal diet; AJT = basal diet + *Lactobacillus plantarum* AP 6-5 (5 × 10^10^ CFU/d); NM = basal diet + *Lactobacillus plantarum* 18-5-5 (5 × 10^10^ CFU/d); LP42 = basal diet + *Lactobacillus plantarum* Y2-2-3 (5 × 10^10^ CFU/d).

AST, aspartate aminotransferase; ALT, alanine aminotransferase; TP, total protein; ALB, albumin; GLOB, globulin; A/G, albumin/globulin ratio; ALP, alkaline phosphatase; GGT, gamma-glutamyl transferase; TBA, total bile acid; CREA, creatinine; GLU, glucose; CO_2_, carbon dioxide; NO, nitric oxide; TCHO, total cholesterol; TRIG, triglycerides; HDL-C, high-density lipoprotein cholesterol; LDL-C, low-density lipoprotein cholesterol.

### 3.5 Alpha and beta diversity of rumen microbiota

**Figure 1:**
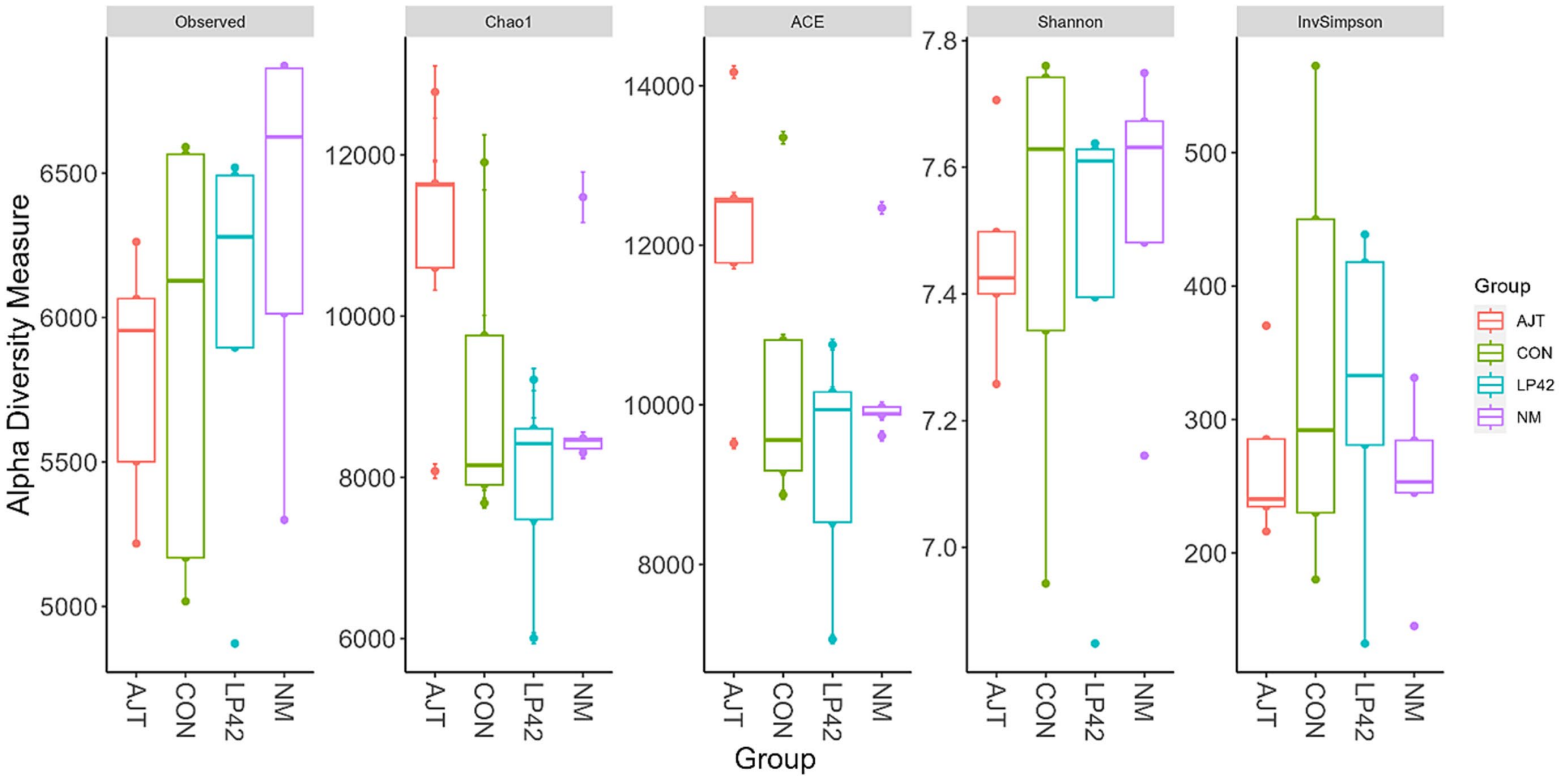
Effects of different *Lactobacillus* strains on the alpha diversity of rumen microbiota in dairy cows.

The effects of the four dietary treatments (CON, AJT, NM, and LP42) on the *α*-diversity of the rumen microbiota were evaluated based on several indices, including Observed OTUs, Chao1, ACE, Shannon, and Inverse Simpson. No significant differences were detected among the groups for any of these indices (*p* > 0.05; Figure 1).

**Figure 2:**
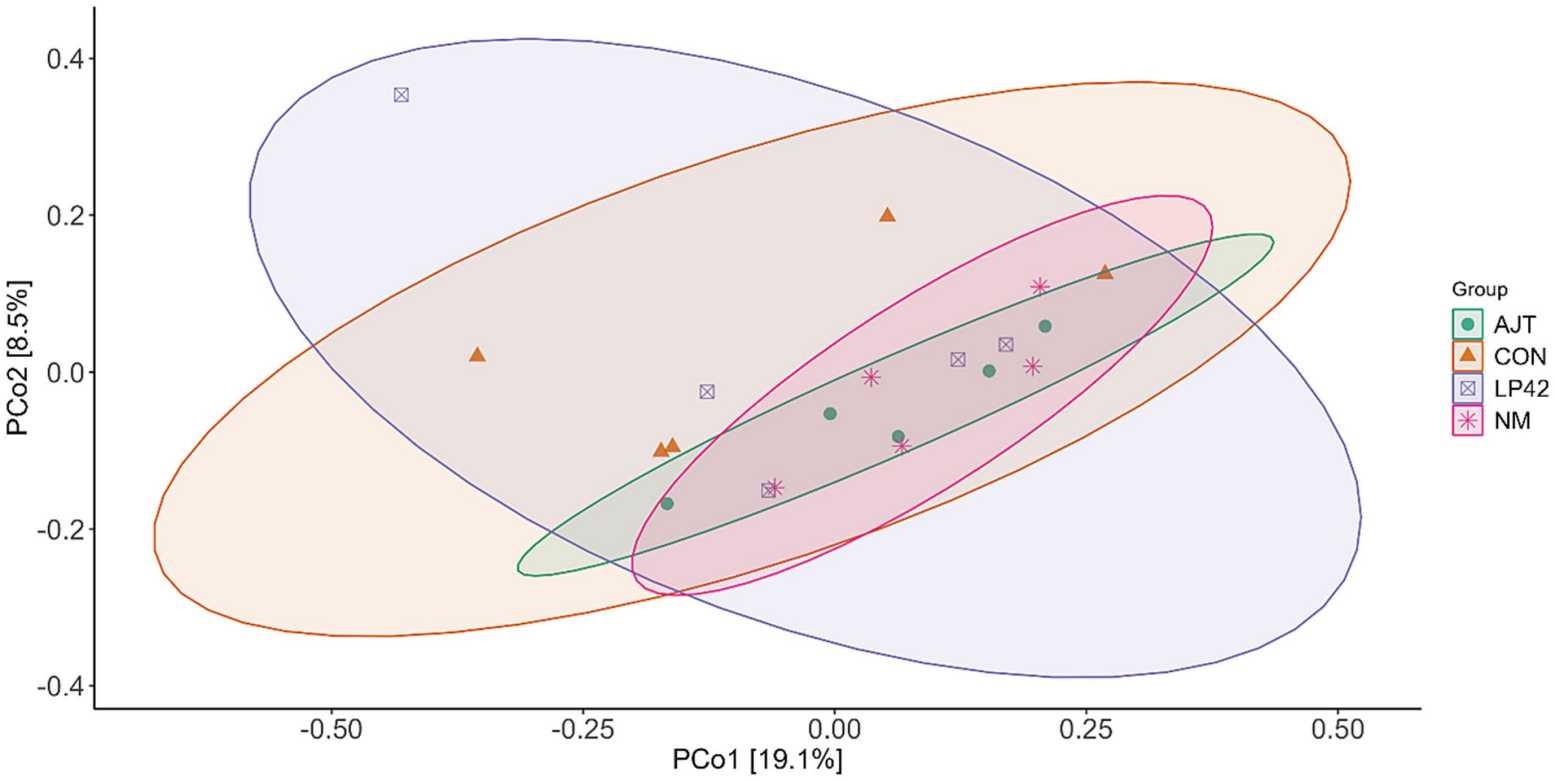
Effects of different *Lactobacillus* strains on the beta diversity of rumen microbiota in dairy cows. PCoA based on Bray–Curtis distance was performed to visualize differences in rumen bacterial communities among dietary groups. Statistical differences were evaluated using PERMANOVA (Permutational Multivariate Analysis of Variance).

The *β*-diversity of the rumen microbiota was evaluated using Bray–Curtis distance metrics. PCoA revealed that the CON group exhibited a wider dispersion compared with the other three groups, indicating greater variability in microbial community composition. Axis 1 and Axis 2 accounted for 19.1 and 8.5% of the total variation, respectively (Figure 2). However, PERMANOVA analysis showed no significant differences in microbial structure among the groups (*p* = 0.476).

### 3.6 Rumen microbiota composition and function

**Figure 3:**
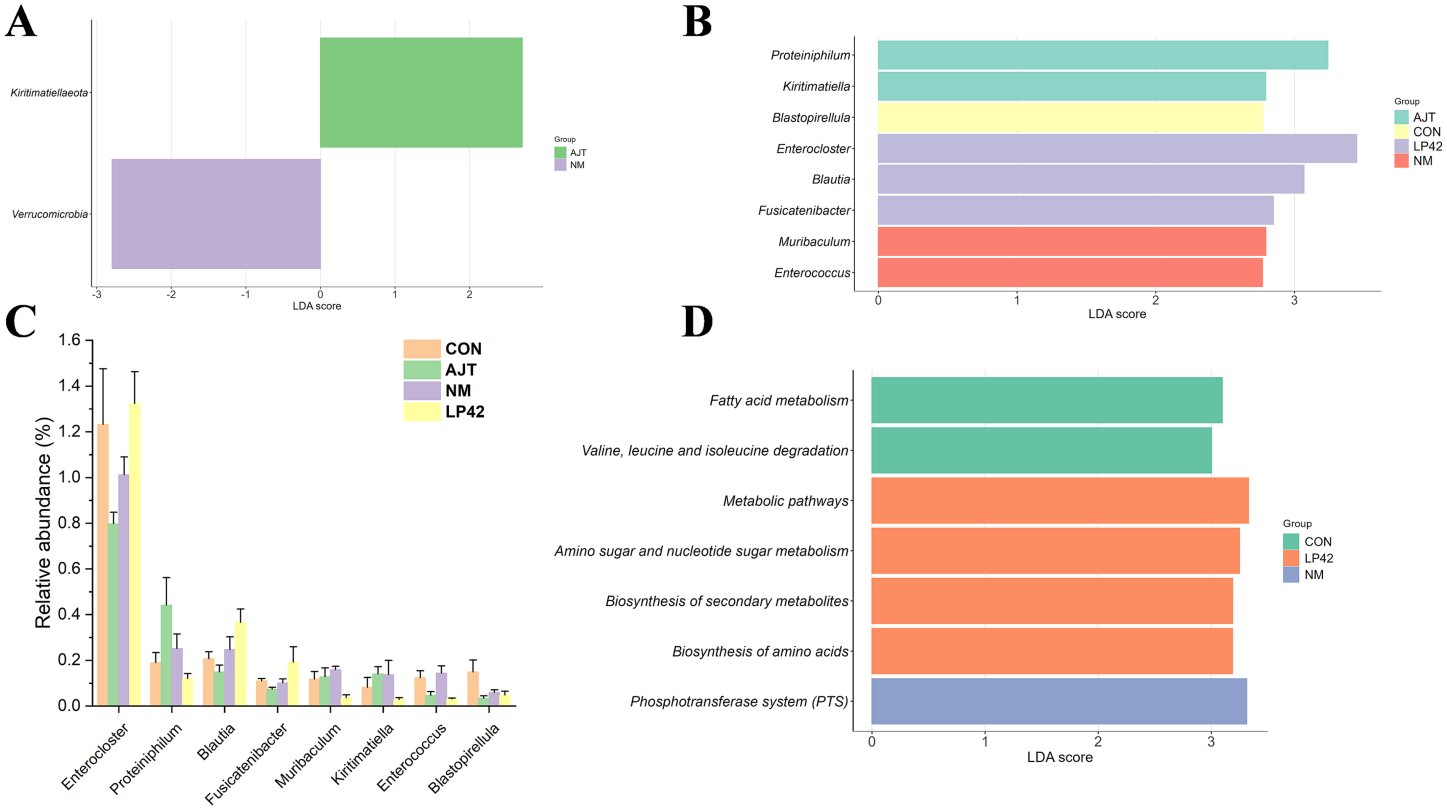
Effects of different *Lactobacillus* strains on the composition and function of the rumen microbiota in dairy cows. **(A)** LEfSe analysis showing enriched bacterial phyla in the rumen (*p* < 0.05, LDA ≥ 2.7). **(B)** LEfSe analysis showing enriched bacterial genera in the rumen (*p* < 0.05, LDA ≥ 2.5). **(C)** Representative bacterial genera significantly affected in the rumen. **(D)** LEfSe analysis showing enriched KEGG pathways (*p* < 0.05, LDA > 3.0).

LEfSe analysis revealed distinct differences in the rumen microbiota composition among treatments. At the phylum level, *Verrucomicrobia* and *Kiritimatiellaeota* were significantly enriched in the NM and AJT groups, respectively (*p* < 0.05, LDA ≥ 2.5; Figure 3A). At the genus level, the LP42 group was enriched with *Enterocloster, Acetobacter*, unclassified Firmicutes, *Blautia*, and *Fusicatenibacter*, whereas *Proteiniphilum* and *Kiritimatiella* were predominant in the AJT group. The NM group showed enrichment of *Muribaculum* and *Enterococcus*, while the CON group was characterized by a higher abundance of *Blastopirellula* (Figure 3B). The relative abundances of the major bacterial genera are shown in Figure 3C.

Functional prediction based on KEGG pathway analysis identified seven significantly enriched pathways (*p* < 0.05, LDA ≥ 3.0; Figure 3D) among the CON, LP42, and NM groups. In the NM group, only the PTS pathway was significantly enriched, whereas the LP42 group exhibited upregulation of Metabolic pathways, Biosynthesis of amino acids, Biosynthesis of secondary metabolites, and Amino sugar and nucleotide sugar metabolism. The CON group was mainly associated with Fatty acid metabolism and Valine, leucine and isoleucine degradation. No KEGG genes were significantly enriched (*p* > 0.05, LDA < 3.0).

## 4 Discussion

In this study, both *in vitro* and *in vivo* experiments were conducted to evaluate the effects of three *Lactobacillus plantarum* strains (18-5-5, AP 6-5, and Y2-2-3) on rumen fermentation, microbial protein synthesis, and production performance. In the *in vitro* experiment, lactic acid concentration was higher in the CON and LP42 treatments compared to NM and AJT, while microbial protein (MCP) levels were higher in CON, AJT, and LP42 than in NM. Accumulation of lactic acid can lower rumen pH, inhibiting fiber degradation (Plaizier et al., 2008) and potentially reducing energy efficiency (Krause and Oetzel, 2006). Additionally, acetate concentration decreased in all *Lactobacillus*-treated groups compared to the control, reflecting short-term shifts in substrate utilization under *in vitro* conditions. MCP is a key source of high-quality protein for the host, and higher MCP levels are generally associated with increased milk protein content (Dewhurst et al., 2000); however, no significant differences in milk protein were observed in the *in vivo* feeding experiment, indicating that the short-term in vitro changes did not fully translate to production responses *in vivo*.

**Figure 4:**
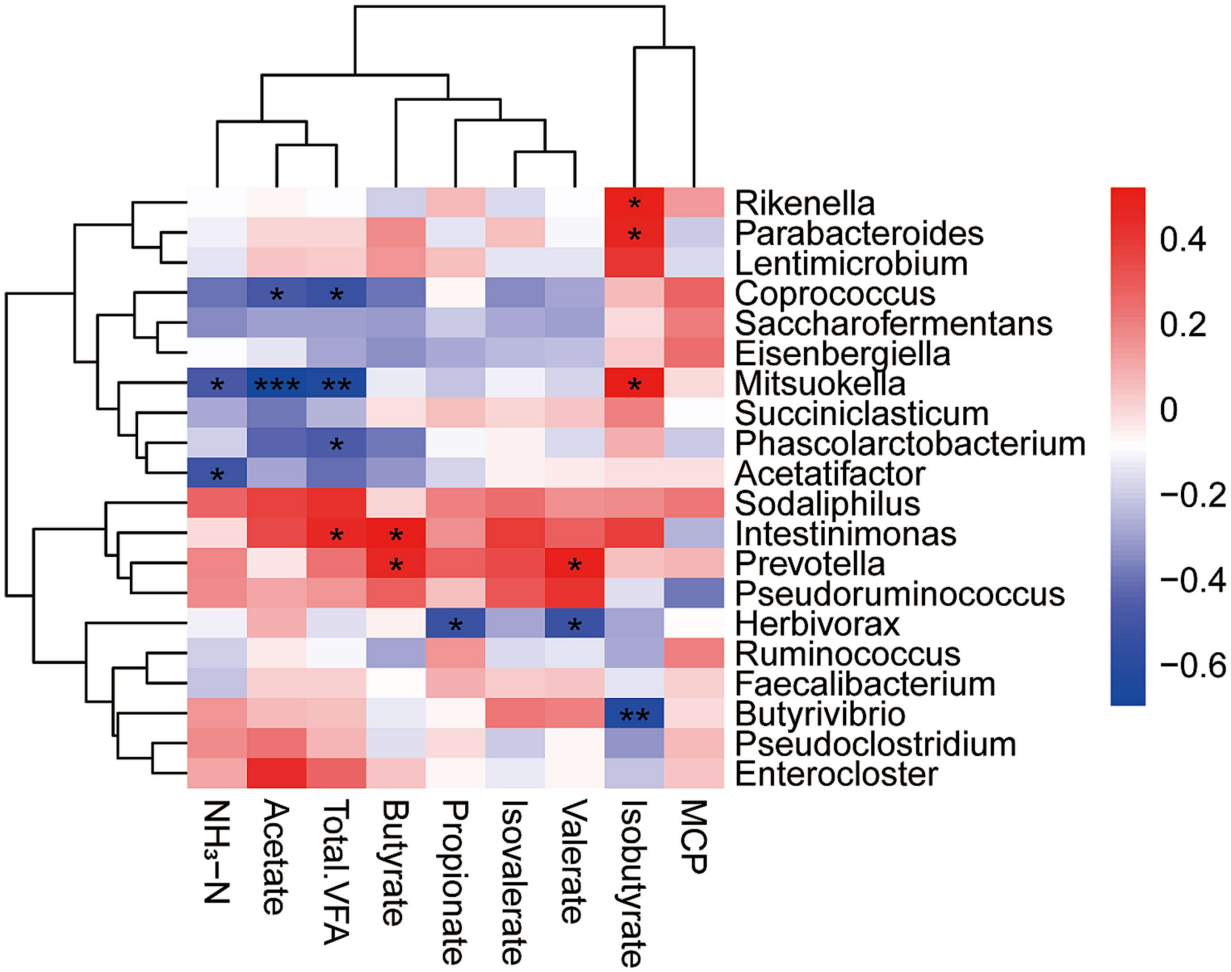
XXX.

In the *in vivo* experiment, ruminal valeric acid concentration was significantly affected by dietary treatment, with LP42 showing the highest level, significantly higher than CON and NM. Valeric acid has been reported to stimulate microbial protein synthesis (Piva et al., 1988), and increased ruminal concentrations of valerate and isovalerate promote MCP production *in vitro* (Piva et al., 1988). Despite these changes, rumen pH remained stable across all groups, while faecal pH was significantly higher in NM and LP42, suggesting enhanced buffering or microbial activity in the hindgut. Notably, NM also exhibited the highest DMI, milk yield, and lactose concentration. As lactose is the main carbohydrate in milk and determines milk osmotic pressure, increased lactose content can directly drive higher milk production (Hettinga, 2019). Higher DMI enables cows to better meet their nutrient requirements, further supporting increased milk yield (Allen, 2000; Beauchemin and McGinn, 2006; McNamara et al., 2003). Compared with the treatment groups, the CON group had higher milk fat and total solids, likely due to a dilution effect caused by increased milk yield rather than as a direct consequence of the changes in ruminal acetate observed in vitro (Bauman and Griinari, 2003). Together, these findings indicate that *Lactobacillus* supplementation can modulate microbial activity and fermentation patterns, with subsequent effects on milk yield and composition, although in vitro changes do not always directly predict *in vivo* production responses (Figure 4).

The results of blood biochemical indices in this study showed that cows in the AJT group had the highest total cholesterol (TCHO) concentration, significantly higher than the CON and NM groups. Similarly, the AJT group exhibited the highest HDL-C concentration, followed by LP42, with both values being significantly higher than those in CON and NM. Serum HDL-C levels in dairy cows are often associated with reproductive performance and milk yield (Duran et al., 2021). Both TCHO and HDL-C are key indicators of fat metabolism, and higher TCHO has been reported to support milk fat synthesis and potentially enhance milk production (Bernal-Santos et al., 2003). These findings are consistent with previous studies indicating that certain *Lactobacillus* strains can improve lipid metabolism and may have beneficial effects on cardiovascular health (Ooi and Liong, 2010).

In this study, 16S rRNA sequencing revealed that dietary supplementation with the three *Lactobacillus* strains altered rumen microbial composition, with ten bacterial genera and two phyla showing enrichment. At the phylum level, *Verrucomicrobia* was enriched in the NM group, while *Kiritimatiellaeota* was enriched in the AJT group. Both phyla are known to participate in the degradation of complex polysaccharides, producing SCFAs such as acetate, propionate, and butyrate, which are crucial for rumen function and provide energy for milk synthesis (Henderson et al., 2015; Yang et al., 2023). At the genus level, the LP42 group showed enrichment in *Enterocloster*, *Acetobacter*, *Unclassified Firmicutes*, *Blautia*, and *Fusicatenibacter*, all associated with fiber digestion and acetate production (Bauman and Griinari, 2003; Jami et al., 2014; Stewart et al., 1997). This suggests that LP42 supplementation may enhance acetate availability to support milk fat synthesis. In the AJT group, *Proteiniphilum* and *Kiritimatiella* were enriched, with reported roles in enhancing ruminal fermentation and fatty acid biosynthesis (Kim et al., 2020; Yang et al., 2023). The NM group was enriched in *Muribaculum* and *Enterococcus*, which could improve fiber degradation, provide energy substrates for microbial growth and host absorption, and contribute to CLA synthesis (Zhu et al., 2024; Abedini et al., 2023). In contrast, the CON group showed enrichment only in *Blastopirellula*, a fiber-digesting genus (Jami et al., 2014).

Functional predictions further highlighted strain-specific effects. The NM group exhibited upregulation of the phosphotransferase system (PTS), facilitating sugar uptake and phosphorylation, which may underlie the higher lactose content and milk yield observed (Martin and Russell, 1986; Lin et al., 2016; Nafikov and Beitz, 2007). In the LP42 group, pathways related to amino acid and nucleotide sugar metabolism were upregulated, potentially enhancing microbial protein synthesis and corroborating the increased MCP observed *in vitro* (Amin et al., 2022). The CON group showed upregulation of fatty acid and branched-chain amino acid degradation pathways, which aligns with the higher milk fat content recorded. Overall, these results indicate that dietary inclusion of specific *Lactobacillus* strains can selectively enrich fiber-digesting and metabolically active microbes, modulate functional pathways related to carbohydrate and protein metabolism, and contribute to differences in milk production and composition.

## 5 Conclusion

This study demonstrates that dietary supplementation with different *Lactobacillus plantarum* strains can modulate rumen fermentation, alter rumen microbiota composition, and improve milk yield and composition in lactating Holstein cows. Notably, supplementation with strain 18-5-5 resulted in the highest dry matter intake and milk yield, accompanied by increased lactose content. Functional predictions of KEGG pathways support these observations, showing enrichment in sugar transport and phosphorylation pathways in the NM group. These findings highlight the potential of targeted probiotic supplementation to enhance dairy cow performance and rumen health, offering a strategy for more efficient and sustainable milk production.

## Data Availability

The data presented in this study are available for public access. Relevant data can be found at the following website: https://www.ncbi.nlm.nih.gov, accession number PRJNA1429271.

## References

[ref1] Abedini R. Zaghari G. Jabbari L. Salekdeh G. H. Hashemi M. (2023). A potential probiotic *Enterococcus faecium* isolated from camel rumen, fatty acids biotransformation, antilisteria activity and safety assessment. Int. Dairy J. 145:105706. doi: 10.1016/j.idairyj.2023.105706

[ref2] Allen M. S. (2000). Effects of diet on short-term regulation of feed intake by lactating dairy cattle. J. Dairy Sci. 83, 1598–1624. doi: 10.3168/jds.S0022-0302(00)75030-2, 10908065

[ref3] Amin A. B. Zhang L. Zhang J. Mao S. (2022). Fermented soybean meal modified the rumen microbiome to enhance the yield of milk components in Holstein cows. Appl. Microbiol. Biotechnol. 106, 7627–7642. doi: 10.1007/s00253-022-12240-2, 36264306

[ref5] AOAC. (2005). Official Methods of Analysis of AOAC. Rockville, MD: AOAC International.

[ref6] Bauman D. E. Griinari J. M. (2003). Nutritional regulation of milk fat synthesis. Annu. Rev. Nutr. 23, 203–227. doi: 10.1146/annurev.nutr.23.011702.073408, 12626693

[ref7] Beauchemin K. A. McGinn S. M. (2006). Methane emissions from beef cattle: effects of fumaric acid, essential oil, and canola oil. J. Anim. Sci. 84, 1489–1496. doi: 10.2527/2006.8461489x, 16699105

[ref8] Bernal-Santos G. Perfield J. W. Barbano D. M. Bauman D. E. Overton T. R. (2003). Production responses of dairy cows to dietary supplementation with conjugated linoleic acid (CLA) during the transition period and early lactation. J. Dairy Sci. 86, 3218–3228. doi: 10.3168/jds.S0022-0302(03)73925-3, 14594242

[ref9] Bolger A. M. Lohse M. Usadel B. (2014). Trimmomatic: a flexible trimmer for Illumina sequence data. Bioinformatics 30, 2114–2120. doi: 10.1093/bioinformatics/btu170, 24695404 PMC4103590

[ref10] Boyd J. West J. W. Bernard J. K. (2011). Effects of the addition of direct-fed microbials and glycerol to the diet of lactating dairy cows on milk yield and apparent efficiency of yield. J. Dairy Sci. 94, 4616–4622. doi: 10.3168/jds.2010-3984, 21854934

[ref12] Campana R. van Hemert S. Baffone W. (2017). Strain-specific probiotic properties of lactic acid bacteria and their interference with human intestinal pathogens invasion. Gut Pathog. 9:12. doi: 10.1186/s13099-017-0162-4, 28286570 PMC5338089

[ref13] Caporaso J. G. Kuczynski J. Stombaugh J. Bittinger K. Bushman F. D. Costello E. K. . (2010). QIIME allows analysis of high-throughput community sequencing data. Nat. Methods 7, 335–336. doi: 10.1038/nmeth.f.303, 20383131 PMC3156573

[ref14] Chaney A. L. Marbach E. P. (1962). Modified reagents for determination of urea and ammonia. Clin. Chem. 8, 130–132. doi: 10.1093/clinchem/8.2.130, 13878063

[ref15] Chaucheyras-Durand F. Durand H. (2010). Probiotics in animal nutrition and health. Benef. Microbes 1, 3–9. doi: 10.3920/bm2008.1002 21840795

[ref16] Dewhurst R. J. Davies D. R. Merry R. J. (2000). Microbial protein supply from the rumen. Anim. Feed Sci. Technol. 85, 1–21. doi: 10.1016/S0377-8401(00)00139-5

[ref17] Duran M. J. Kannampuzha-Francis J. Nydam D. Behling-Kelly E. (2021). Characterization of particle size distribution of plasma lipoproteins in dairy cattle using high-resolution polyacrylamide electrophoresis. Front. Anim. Sci. 2:678085. doi: 10.3389/fanim.2021.678085

[ref18] Edgar R. C. (2013). UPARSE: highly accurate OTU sequences from microbial amplicon reads. Nat. Methods 10:996. doi: 10.1038/nmeth.2604, 23955772

[ref19] Henderson G. Cox F. Ganesh S. Jonker A. Young W. Janssen P. H. (2015). Rumen microbial community composition varies with diet and host, but a core microbiome is found across a wide geographical range. Sci. Rep. 5:14567. doi: 10.1038/srep14567, 26449758 PMC4598811

[ref20] Hettinga K. A. (2019). “Lactose in the dairy production chain” in Lactose (Academic Press), (Wageningen, The Netherlands: Wageningen University & Research) 231–266.

[ref21] Jami E. White B. A. Mizrahi I. (2014). Potential role of the bovine rumen microbiome in modulating milk composition and feed efficiency. PLoS One 9:e85423. doi: 10.1371/journal.pone.0085423, 24465556 PMC3899005

[ref23] Kim S. H. Mamuad L. L. Islam M. Lee S. S. (2020). Reductive acetogens isolated from ruminants and their effect on in vitro methane mitigation and milk performance in Holstein cows. J Anim Sci Technol. 62, 1–13. doi: 10.5187/jast.2020.62.1.1, 32082593 PMC7008121

[ref24] Krause K. M. Oetzel G. R. (2006). Understanding and preventing subacute ruminal acidosis in dairy herds: a review. Anim. Feed Sci. Technol. 126, 215–236. doi: 10.1016/j.anifeedsci.2005.08.004

[ref25] Lin Y. Sun X. Hou X. Qu B. Gao X. Li Q. (2016). Effects of glucose on lactose synthesis in mammary epithelial cells from dairy cow. BMC Vet. Res. 12, 81–81. doi: 10.1186/s12917-016-0704-x, 27229304 PMC4880877

[ref26] Liu C. Cui Y. Li X. Yao M. (2020). Microeco: an R package for data mining in microbial community ecology. FEMS Microbiol. Ecol. 97:fiaa255. doi: 10.1093/femsec/fiaa255 33332530

[ref27] Magoc T. Salzberg S. L. (2011). FLASH: fast length adjustment of short reads to improve genome assemblies. Bioinformatics 27, 2957–2963. doi: 10.1093/bioinformatics/btr507, 21903629 PMC3198573

[ref29] Mao S. Zhang R. Wang D. Zhu W. (2012). The diversity of the fecal bacterial community and its relationship with the concentration of volatile fatty acids in the feces during subacute rumen acidosis in dairy cows. BMC Vet. Res. 8:237. doi: 10.1186/1746-6148-8-237, 23217205 PMC3582618

[ref30] Martin S. A. Russell J. B. (1986). Phosphoenolpyruvate-dependent phosphorylation of hexoses by ruminal bacteria: evidence for the phosphotransferase transport system. Appl. Environ. Microbiol. 52, 1348–1352. doi: 10.1128/aem.52.6.1348-1352.1986, 3789722 PMC239232

[ref31] McNamara S. O’Mara F. P. Rath M. Murphy J. J. (2003). Effects of different transition diets on dry matter intake, milk production, and milk composition in dairy cows. J. Dairy Sci. 86, 2397–2408. doi: 10.3168/jds.S0022-0302(03)73834-X, 12906058

[ref32] Monteiro H. F. Lelis A. L. J. Fan P. Calvo A. B. Lobo R. R. Arce-Cordero J. A. . (2022). Effects of lactic acid-producing bacteria as direct-fed microbials on the ruminal microbiome. J. Dairy Sci. 105, 2242–2255. doi: 10.3168/jds.2021-21025 34998552

[ref33] Monteiro H. F. Paula E. M. Muck R. E. Broderick G. A. Faciola A. P. (2021). Effects of lactic acid bacteria in a silage inoculant on ruminal nutrient digestibility, nitrogen metabolism, and lactation performance of high-producing dairy cows. J. Dairy Sci. 104, 8826–8834. doi: 10.3168/jds.2021-20155, 34053758

[ref34] Nafikov R. A. Beitz D. C. (2007). Carbohydrate and lipid metabolism in farm animals. J. Nutr. 137, 702–705. doi: 10.1093/jn/137.3.702, 17311965

[ref35] NASEM. (2021). Nutrient Requirements of Dairy Cattle: Eighth revised edition. Washington, DC: The National Academies Press. 38386771

[ref36] O'Hara E. Neves A. L. A. Song Y. Guan L. L. (2020). The role of the gut microbiome in cattle production and health: driver or passenger? Annu. Rev. Anim. Biosci. 8, 199–220. doi: 10.1146/annurev-animal-021419-083952, 32069435

[ref37] Ooi L. G. Liong M. T. (2010). Cholesterol-lowering effects of probiotics and prebiotics: a review of in vivo and in vitro findings. Int. J. Mol. Sci. 11, 2499–2522. doi: 10.3390/ijms11062499, 20640165 PMC2904929

[ref38] Philippeau C. Lettat A. Martin C. Silberberg M. Morgavi D. P. Ferlay A. . (2017). Effects of bacterial direct-fed microbials on ruminal characteristics, methane emission, and milk fatty acid composition in cows fed high- or low-starch diets. J. Dairy Sci. 100, 2637–2650. doi: 10.3168/jds.2016-11663, 28161181

[ref39] Piva G. Masoero F. Curto O. (1988). The effect of isoacids on ruminal fermentation: in vitro experiments. Reprod. Nutr. Dev. 28, 163–164.2908189

[ref40] Plaizier J. C. Krause D. O. Gozho G. N. McBride B. W. (2008). Subacute ruminal acidosis in dairy cows: the physiological causes, incidence and consequences. Vet. J. 176, 21–31. doi: 10.1016/j.tvjl.2007.12.016, 18329918

[ref41] Quast C. Pruesse E. Yilmaz P. Gerken J. Schweer T. Yarza P. . (2013). The SILVA ribosomal RNA gene database project: improved data processing and web-based tools. Nucleic Acids Res. 41, D590–D596. doi: 10.1093/nar/gks1219, 23193283 PMC3531112

[ref42] Raeth-Knight M. L. Linn J. G. Jung H. G. (2007). Effect of direct-fed microbials on performance, diet digestibility, and rumen characteristics of Holstein dairy cows. J. Dairy Sci. 90, 1802–1809. doi: 10.3168/jds.2006-643, 17369221

[ref43] Reddy P. R. K. Hyder I. (2023). “Ruminant digestion” in Textbook of veterinary physiology, (Kolkata, India: Department of Veterinary Physiology West Bengal University of Animal and Fishery Sciences) 353–366.

[ref44] Segata N. Izard J. Waldron L. Gevers D. Miropolsky L. Garrett W. S. . (2011). Metagenomic biomarker discovery and explanation. Genome Biol. 12:R60. doi: 10.1186/gb-2011-12-6-r60, 21702898 PMC3218848

[ref45] Shen J. S. Chai Z. Song L. J. Liu J. X. Wu Y. M. (2012). Insertion depth of oral stomach tubes may affect the fermentation parameters of ruminal fluid collected in dairy cows1. J. Dairy Sci. 95, 5978–5984. doi: 10.3168/jds.2012-5499, 22921624

[ref47] Stewart C. S. Flint H. J. Bryant M. P. (1997). “The rumen bacteria” in The rumen microbial ecosystem. eds. Hobson P. N. Stewart C. S. (Dordrecht: Springer Netherlands), 10–72.

[ref48] Theodorou M. K. Williams B. A. Dhanoa M. S. McAllan A. B. France J. (1994). A simple gas production method using a pressure transducer to determine the fermentation kinetics of ruminant feeds. Anim. Feed Sci. Technol. 48, 185–197. doi: 10.1016/0377-8401(94)90171-6

[ref49] Tsai Y.-T. Cheng P.-C. Pan T.-M. (2012). The immunomodulatory effects of lactic acid bacteria for improving immune functions and benefits. Applied Microbiol. Biotechnol. 96, 853–862. doi: 10.1007/s00253-012-4407-3, 23001058

[ref51] Van Keulen J. Young B. A. (1977). Evaluation of acid-insoluble ash as a natural marker in ruminant digestibility studies. J. Anim. Sci. 44, 282–287. doi: 10.2527/jas1977.442282x

[ref52] VandeHaar M. St-Pierre N. (2006). Major advances in nutrition: relevance to the sustainability of the dairy industry. J. Dairy Sci. 89, 1280–1291. doi: 10.3168/jds.S0022-0302(06)72196-8, 16537960

[ref53] Wang J. Ji H. Wang S. Liu H. Zhang W. Zhang D. . (2018). Probiotic *Lactobacillus plantarum* promotes intestinal barrier function by strengthening the epithelium and modulating gut microbiota. Front. Microbiol. 9:1933. doi: 10.3389/fmicb.2018.01953, 30197632 PMC6117384

[ref54] Wemheuer F. Taylor J. A. Daniel R. Johnston E. Meinicke P. Thomas T. . (2020). Tax4Fun2: prediction of habitat-specific functional profiles and functional redundancy based on 16S rRNA gene sequences. Environ. Microbiome. 15:11. doi: 10.1186/s40793-020-00358-7, 33902725 PMC8067651

[ref55] Xie F. Xu L. Wang Y. Mao S. Ishaq S. L. Chen L. . (2021). Metagenomic sequencing reveals that high-grain feeding alters the composition and metabolism of Cecal microbiota and induces Cecal mucosal injury in sheep. Msystems 6, e0091521–e0091521. doi: 10.1128/mSystems.00915-21, 34609166 PMC8547435

[ref56] Yang S. Zheng J. He S. Yuan Z. Wang R. Wu D. (2023). Exploring the elevation dynamics of rumen bacterial communities in barn feeding cattle from 900 to 3,600 meters by full-length 16S sequencing. Front. Vet. Sci. 10:1169573. doi: 10.3389/fvets.2023.1169573, 37533459 PMC10390322

[ref57] Zhang T. Mu Y. Zhang R. Xue Y. (2022). Responsive changes of rumen microbiome and metabolome in dairy cows with different susceptibility to subacute ruminal acidosis. Anim. Nutr. 8, 331–340. doi: 10.1016/j.aninu.2021.10.009, 35024470 PMC8718735

[ref58] Zhu Y. Chen B. Zhang X. Akbar M. T. Wu T. Zhang Y. . (2024). Exploration of the muribaculaceae family in the gut microbiota: diversity, metabolism, and function. Nutrients 16:2660. doi: 10.3390/nu16162660, 39203797 PMC11356848

